# Assessment and validation of the Community Maternal Danger Score algorithm

**DOI:** 10.1186/s41256-022-00240-8

**Published:** 2022-02-11

**Authors:** Rajan Bola, Fanan Ujoh, Ugochinyere Vivian Ukah, Ronald Lett

**Affiliations:** 1 Canadian Network for International Surgery, #212-1650 Duranleau St, Vancouver, BC V6H 3S4 Canada; 2 Centre for Sustainability & Resilient Infrastructure & Communities, London South Bank University, London, UK; 3 Department of Epidemiology, Biostatistics, and Occupational Health, McGill University, Montreal, Canada

**Keywords:** Antenatal care, LMIC, Maternal mortality, Nigeria, Risk analysis, Skilled birth attendants

## Abstract

**Background:**

High rates of maternal mortality in low-and-middle-income countries (LMICs) are associated with the lack of skilled birth attendants (SBAs) at delivery. Risk analysis tools may be useful to identify pregnant women who are at risk of mortality in LMICs. We sought to develop and validate a low-cost maternal risk tool, the Community Maternal Danger Score (CMDS), which is designed to identify pregnant women who need an SBA at delivery.

**Methods:**

To design the CMDS algorithm, an initial scoping review was conducted to identify predictors of the need for an SBA. Medical records of women who delivered at the Federal Medical Centre in Makurdi, Nigeria (2019–2020) were examined for predictors identified from the literature review. Outcomes associated with the need for an SBA were recorded: caesarean section, postpartum hemorrhage, eclampsia, and sepsis. A maternal mortality ratio (MMR) was determined. Multivariate logistic regression analysis and area under the curve (AUC) were used to assess the predictive ability of the CMDS algorithm.

**Results:**

Seven factors from the literature predicted the need for an SBA: age (under 20 years of age or 35 and older), parity (nulliparity or grand-multiparity), BMI (underweight or overweight), fundal height (less than 35 cm or 40 cm and over), adverse obstetrical history, signs of pre-eclampsia, and co-existing medical conditions. These factors were recorded in 589 women of whom 67% required an SBA (n = 396) and 1% died (n = 7). The MMR was 1189 per 100,000 (95% CI 478–2449). Signs of pre-eclampsia, obstetrical history, and co-existing conditions were associated with the need for an SBA. Age was found to interact with parity, suggesting that the CMDS requires adjustment to indicate higher risk among younger multigravida and older primigravida women. The CMDS algorithm had an AUC of 0.73 (95% CI 0.69–0.77) for predicting whether women required an SBA, and an AUC of 0.85 (95% CI 0.67–1.00) for in-hospital mortality.

**Conclusions:**

The CMDS is a low-cost evidence-based tool that uses 7 risk factors assessed on 589 women from Makurdi. Non-specialist health workers can use the CMDS to standardize assessment and encourage pregnant women to seek an SBA in preparation for delivery, thus improving care in countries with high rates of maternal mortality.

## Background

Reports from the World Health Organization (WHO) indicate that sub-Saharan Africa accounts for two-thirds of global maternal deaths [1]. Similarly, studies reveal that a Nigerian woman's chance of dying from pregnancy and childbirth is 1 in 22, in contrast with 1 in 4900 among women in developed nations [2]. As of 2017, Nigeria’s population amounted to only 2% of the world population, yet the country accounted for 23% of the global maternal death burden, with a maternal mortality ratio (MMR) of 917 deaths per 100,000 live births. [1, 3]

Benue State, which is one of the 36 states of Nigeria, also has a large proportion of women at risk for maternal mortality and morbidity [4, 5]. Studies reveal that access to skilled birth attendants (SBAs) is limited in Benue State [4–7]. No MMR reports for Benue State have been published since 2018 [5], despite there being a critical need for data to inform antenatal care and delivery services within the state. [4]

Global efforts have been channelled towards reducing maternal mortality and morbidity across heavily impacted countries, such as Nigeria. One of such efforts is enshrined in the United Nations Sustainable Development Goal Number 3, Targets 3.1, 3.2, and 3.3 which focus on the reduction of maternal mortality to below 70 per 100,000 live births; eradication of preventable deaths of newborns and under five children; and reduction of premature mortality from non-communicable diseases, respectively, by year 2030 [8]. Studies show that a strong focus on training, recruiting, and supporting SBAs has successfully reduced maternal mortality in countries with a high burden of maternal death. [9, 10].

Interventions targeting maternal and neonatal mortality in parts of Africa present platforms for healthcare workers to support pregnant mothers with valuable information for safer pregnancies and deliveries. Midwives and community health workers in rural regions of Nigeria are in critical shortage and are often the primary healthcare workers conducting obstetrical assessments of pregnant women in care centres [11, 12]. To support these crucial workers, and others providing obstetrical care, the WHO has compiled best-practice guidelines on managing and maintaining maternal health in the prenatal, perinatal, and postnatal periods [13]. However, these recommendations do not allow for a direct estimation of risk, nor do they consider the contextual limitations of maternal care in low-resource settings. Similarly, there are niche scoring systems that aim to prevent maternal mortality by targeting women with risk indicators for specific conditions like pre-eclampsia [14] and near-miss morbidity [15]. However, there exists no comprehensive, validated risk-scoring systems for pregnant women in developing countries.

The community maternal danger score (CMDS) is a novel, low-cost maternal risk analysis tool developed from evidence-based research. The purpose of the CMDS is to improve care delivered by healthcare workers by providing informed and valuable information to pregnant mothers in remote areas where access to specialized medical care is severely limited. The study objective is to validate the CMDS algorithm on a cohort of pregnant women who delivered at a tertiary-hospital in Benue State, Nigeria.

## Methods

### Search strategy

An initial scoping review using Ovid MEDLINE and PubMed was performed between December 2018 to February 2019 to understand the state of knowledge surrounding risk factors that predict poor maternal health outcomes, and in particular, outcomes that can be prevented by a skilled birth attendant. The framework used to appraise the literature was based on a priori knowledge of maternal risk factors in low-resource settings.

### Study inclusion and exclusion criteria

We did not impose any restrictions on study type. The scoping review allowed for the identification of studies that support or introduce new findings within the framework of maternal risk factors and care, and were not restricted to any single region to promote generalizability.

### Data extraction

The articles from this review each had three key elements extracted: the type of risk factor described, the strength of association between the risk factor and maternal mortality or morbidity, and study limitations. The relevant findings from this extract were then used to inform the feasibility of the CMDS algorithm by comparing resource access and obstetrical knowledge in settings where the CMDS was to be implemented.

### Predictive accuracy assessment

The predictive accuracy assessment was conducted using antenatal data from consecutive pregnant women who delivered at the Federal Medical Centre in Makurdi, Nigeria between 2019 and 2020. Makurdi is the capital of Benue State, located in north-central Nigeria. Medical records outlining the visit details of pregnant women were examined retrospectively to record data pertaining to demographics, obstetrical history, medication administration, surgical interventions, and maternal outcomes. This information was abstracted by trained study personnel who inputted the data on an Excel spreadsheet for preliminary analysis, visualization, and data cleaning.

### Statistical analysis

Basic descriptive statistics were used to describe the frequencies of select variables. We calculated a maternal mortality ratio and associated confidence intervals per 100,000 using the binomial method [16]. We used logistic regression modelling to evaluate the predictive ability of risk indicators identified from the literature. The primary outcome of interest was the need for an SBA, which is defined by a composite measure of the patient’s clinical presentation and accompanying symptoms and interventions. These are caesarean section, postpartum hemorrhage (defined by blood loss of 500 mL or more), eclampsia (defined by the need for antihypertensive medications and/or convulsions during birth), and sepsis. A patient who required any of these interventions or presented with either of these complications was designated as needing an SBA. The secondary outcome of interest was in-hospital mortality.

We use RStudio for all statistical analysis (Version 1.4; Vienna, Austria; 2021). Receiver operating curve (ROC) analysis was used to calculate the area under the receiver operating curve (AUC). The AUC was used to inform the discrimination of the CMDS. The AUC assesses the model sensitivity and specificity by computing the area under the ROC. The null model would be a model with an AUC of 0.5 meaning discriminative ability no better than by chance [17]. The closer to 1.0 the model AUC is, the better it performs. An AUC of 0.7 would suggest moderate discrimination, 0.8 would suggest good discrimination, and 0.9 or higher would suggest excellent discrimination. A significant result was defined at 95% with a p-value estimated below 0.05.

### Ethical considerations

Approval to conduct this study was obtained from the Ministry of Health and Human Services, Benue State, Nigeria (FMH/FMC/MED.108/VOL.I/X). A stipulation of the ethical clearance allowed for informed consent to be waived as this study examined medical records and did not require patient interaction. Patient’s anonymity and confidentiality was protected for the duration of this study.

## Results

### CMDS domains

**Fig. 1:**
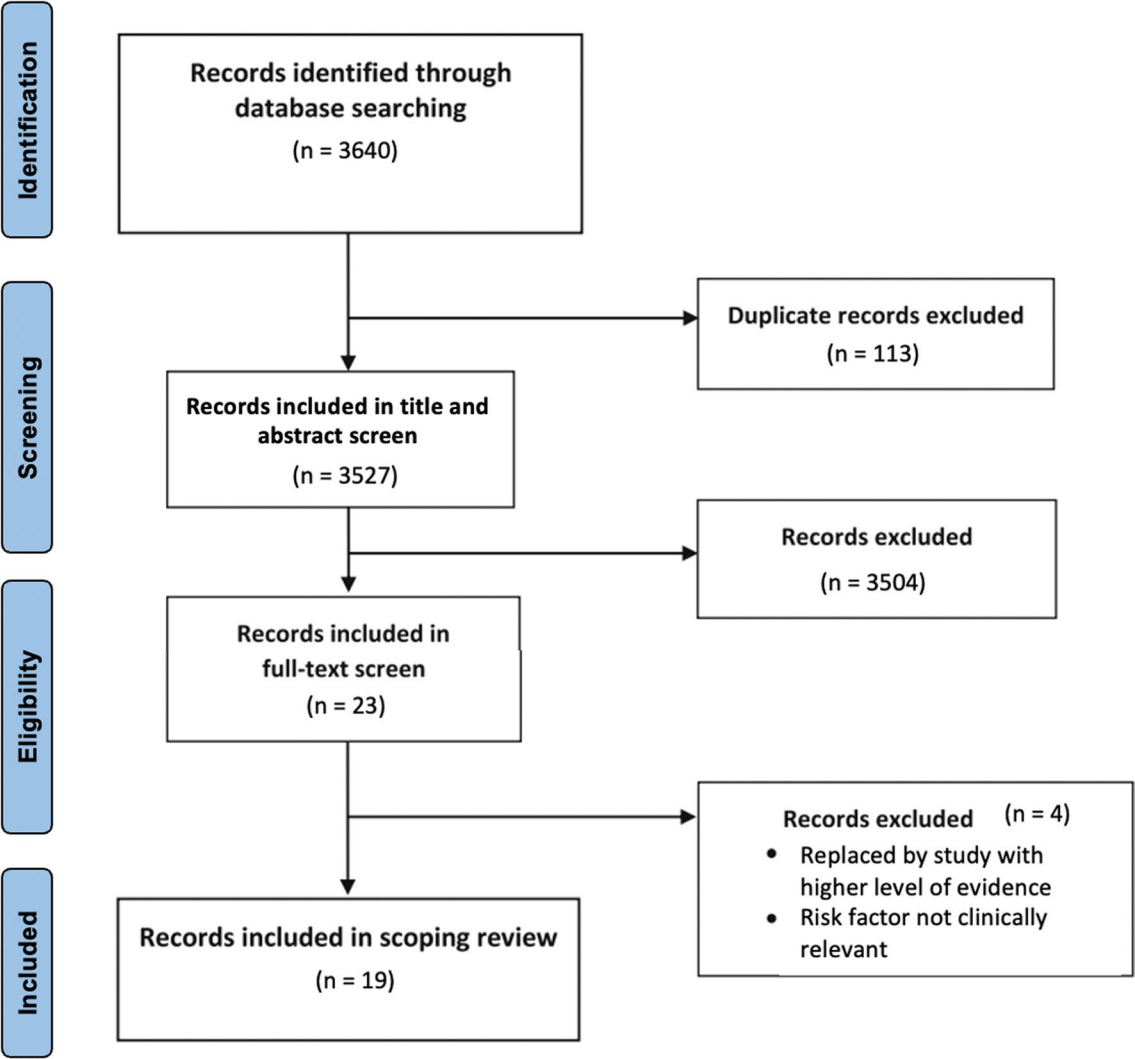
Flow diagram for a scoping review of maternal risk factors examined to create the Community Maternal Danger Score

After the removal of 113 duplicate records, the literature search yielded 3527 studies. 3504 studies were excluded during title and abstract screening, leaving 23 records for full-text screening. After reviewing the full text for these 23 studies, 19 papers were included (Fig. 1).

In total, 7 factors associated with high risk of maternal mortality or morbidity were identified in the literature and included in the CMDS algorithm. The factors include age, parity, patient size, obstetrical history, fundal height, signs of pre-eclampsia, and co-existing conditions (Table 1). The highest risk groups within these factors are women below 20 years of age and older than 35 [18], nulliparous (first pregnancy) or grand multiparous (parity > 4) [19], underweight (body-mass index (BMI) < 18.5 kg/m^2^) or overweight (> 30 kg/m^2^) [20, 21], or have a symphysis-fundal height of less than 35 cm or greater than 40 cm at the third trimester [22, 23]. Furthermore, patients with an obstetrical history of any of: previous hemorrhage [24], previous stillbirth or miscarriage [25], previous breech delivery [26], previous twins (or more) [27], previous pregnancy within 1.5 years or greater than 5 years ago [28], and reported reduction in fetal movements [22] were grouped as: high risk (1 of these conditions) or extremely high risk (2 or more of these conditions). Co-existing conditions (maternal sepsis [29], fever with ruptured membranes [30], human immunodeficiency virus (HIV) [24, 31], anemia [31], tuberculosis [31], female genital mutilation [32, 33], diabetes [29], and malaria [23, 31]) were also recorded on 2-levels (high risk and extremely high risk) based on the number and type of conditions the woman presented with (Table 1). Pre-eclampsia is a common cause of maternal mortality [34] and is classified as a risk factor based on two findings: by the mother’s blood pressure and by other signs that could indicate this condition [22, 35]. Blood pressure is categorized as either normal (< 120 systolic and < 90 diastolic mmHg), high (between 120–140 systolic and 90–100 diastolic mmHg), or very high (> 140 systolic and > 100 diastolic mmHg). Signs of pre-eclampsia include proteinuria, headache, epigastric pain, blurred vision, excessive weight gain of 1 kg or more per week, and seizures. Pre-eclampsia risk was recorded on 2-levels (high risk and extremely high risk) based on a combination of the woman’s blood pressure and signs (Table 1).

**Table 1:** Summary of the 7 domains of the CMDS, including risk-definitions for extremely high risk, high risk, and low risk women

Name of risk factor	Definition for women at extremely high risk, if applicable	Definition for women at high risk	Definition for women at low risk
Age	N/A	Below 20 years of age	20 years of age or older and below 35 years of age
		35 years of age or older	
Parity	N/A	Nulliparous (para = 1)	Multiparous (para 1–4)
		Grand-multiparous (para > 4)	
Patient size	N/A	Underweight (BMI ≤ 18.5 kg/m	Normal weight (BMI > 18.5 and ≤ 30.0 kg/m
		Overweight (BMI > 30.0 kg/m	
Obstetrical history	Two of the following:	One of the following:	Lack of these conditions
	Previous hemorrhage	Previous hemorrhage	
	Previous stillbirth or miscarriage	Previous stillbirth or miscarriage	
	Previous breech delivery	Previous breech delivery	
	Previous twins (or more)	Previous twins (or more)	
	Previous pregnancy within 1.5 years or greater than 5 years ago	Previous pregnancy within 1.5 years or greater than 5 years ago	
	Reported reduction in fetal movements	Reported reduction in fetal movements	
Fundal height (3rd trimester)	N/A	Fundal height ≤ 35 cm	Fundal height > 35 cm and ≤ 40 cm
		Fundal height > 40 cm	
Signs of pre-eclampsia	Very high blood pressure (≥ 140 systolic and ≥ 100 diastolic mmHg)	High blood pressure (> 120 systolic or > 90 diastolic mmHg)	Normal blood pressure (≤ 120 systolic and ≤ 90 diastolic mmHg)
			Lack of the aforementioned signs
	OR		
	Any sign of pre-eclampsia: proteinuria, headache, epigastric pain, blurred vision, excessive weight gain of 1 kg or greater per week, or seizures		
	AND		
	High blood pressure (> 120 systolic or > 90 diastolic mmHg)		
Co-existing conditions	Two of the following:	One of the following:	Lack of the aforementioned conditions
	HIV	HIV	
	Anemia	Anemia	
	Tuberculosis	Tuberculosis	
	Female genital mutilation	Female genital mutilation	
	Diabetes	Diabetes	
	Malaria	Malaria	
	OR		
	One of the following:		
	Maternal sepsis		
	Fever with ruptured membranes		

### Univariate summary of patients

A total of 589 women were included in this study. An SBA was required by 396 women (67.2%) and 1.2% (n = 7) of women experienced a hospital death. The maternal mortality ratio was calculated as 1189 per 100,000 (95% confidence interval (95%CI): 478–2449 per 100,000). The distribution of women matching the risk-definition for each of the CMDS factors is illustrated in Table 2. Only select few women (< 10%) met the risk definition for extremely high-risk categories of signs of obstetrical history, pre-eclampsia, and co-existing conditions.

**Table 2:** Frequency distribution of the 7 risk factors included in the CMDS based on 589 women who delivered at the Federal Medical Centre, Makurdi, Nigeria

Name of risk factor	Number of women meeting risk-definition (out of 589)	Proportion of women meeting risk-definition
SBA (outcome)	n = 396	67.2%
Age: high risk	n = 69	11.7%
Parity: high risk	n = 227	38.5%
Patient size: high risk	n = 83	14.1%
Obstetrical history: high risk	n = 219	37.2%
Obstetrical history: extremely high risk	n = 28	4.8%
Fundal height (3rd trimester)	n = 180	30.6%
Signs of pre-eclampsia: high risk	n = 129	21.9%
Signs of pre-eclampsia: extremely high risk	n = 29	4.9%
Co-existing conditions: high risk	n = 124	21.1%
Co-existing conditions: extremely high risk	n = 39	6.6%

### Predictive accuracy using hospital-based data

The odds ratios for needing an SBA at delivery was greater than 1.0 for all risk factors in the adjusted model, with the exception of fundal height (adjusted odds ratio of 0.697, 95% CI 0.463–1.051) (Table 3). Of the risk factors included in the regression model, only pre-eclampsia, co-existing conditions, and obstetrical history had odds ratios that were statistically significant (Table 3). The inclusion of an interaction term between age and parity demonstrated the need to account for effect modification between these variables and the need for an SBA. Odds ratios for age, parity, patient size, and fundal height, were not found to be statistically significant.

**Table 3:** Multivariate logistic regression analysis for the risk factors included in the CMDS with the need for a skilled birth attendant as the outcome

Name of risk factor	Adjusted odds ratios	95% confidence interval: lower bound	95% confidence interval: upper bound	P-value (*marks a significant result)
Age: high risk	1.827	0.876	3.766	0.103
Parity: high risk	2.846	0.820	11.782	0.117
Patient size: high risk	1.085	0.619	1.869	0.772
Obstetrical history: high risk	2.691	1.770	4.153	< 0.0001^*^
Obstetrical history: extremely high risk	6.819	2.171	30.333	0.003^*^
Fundal height (3rd trimester)	0.697	0.463	1.051	0.084
Signs of Pre-eclampsia: high risk	2.728	1.676	4.572	< 0.0001^*^
Signs of pre-eclampsia: extremely high risk	18.443	3.774	333.322	0.005^*^
Co-existing conditions: high risk	2.092	1.274	3.530	0.004^*^
Co-existing conditions: extremely high risk	3.516	1.402	10.745	0.014^*^
Age and parity (interaction)	0.155	0.038	0.627	0.008^*^

**Fig. 2:**
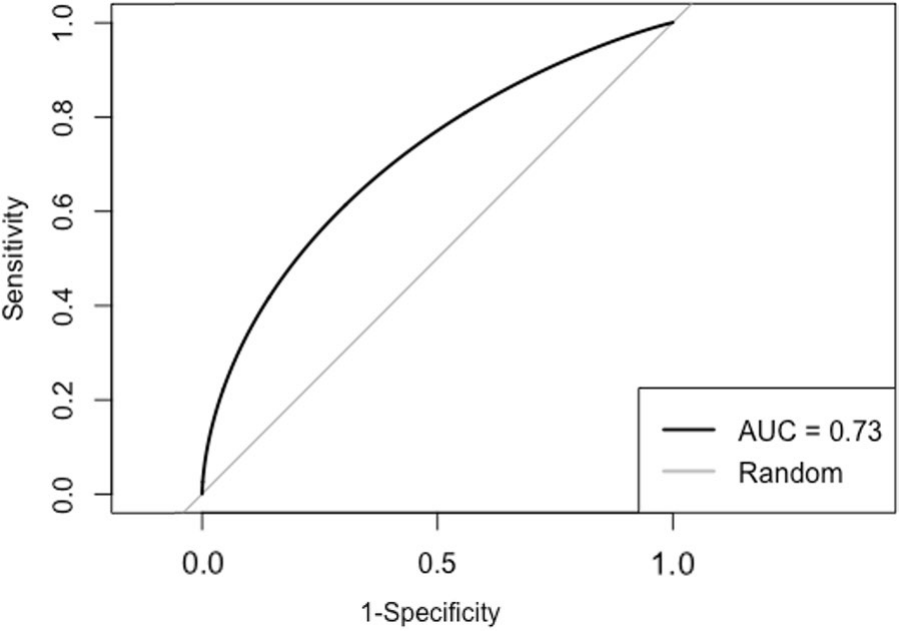
Receiver operating curve and area under the curve for the CMDS with the need for a skilled birth attendant as the outcome

**Fig. 3:**
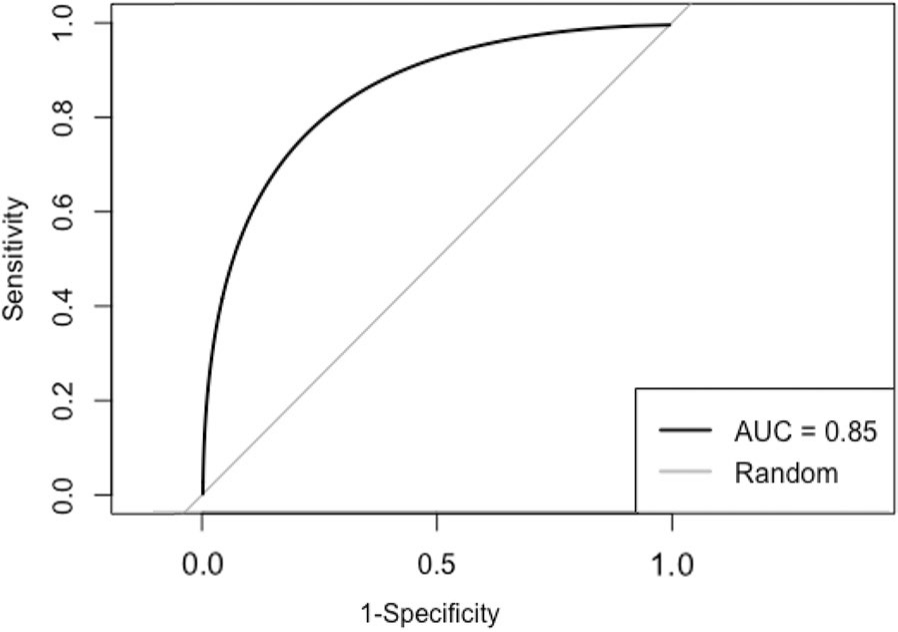
Receiver operating curve and area under the curve for the CMDS with mortality in-hospital as the outcome

The CMDS was moderately discriminative of the need for an SBA during delivery, as indicated by the ROC analysis providing an AUC of 0.73 (95% CI 0.69–0.77) (Fig. 2). When the same model was tested using an outcome based on mortality only, the AUC was 0.85 (95% CI 0.67–1.0) suggesting high discriminative ability (Fig. 3). The adjusted odds ratios were above 1.0 for age, extremely high risk from co-existing conditions, and high risk from pre-eclampsia (Table 4). The remaining odds ratios were below 1.0 and non-significant.

**Table 4:** Multivariate logistic regression analysis for the risk factors included in the CMDS with mortality in-hospital as the outcome

Name of risk factor	Adjusted odds ratios	95% confidence interval: lower bound	95% confidence interval: upper bound	P-value (*marks a significant result)
Age: high risk	1.323	0.001	8.960	0.806
Parity: high risk	0.631	0.066	3.608	0.581
Patient size: high risk	3.675E-08	N/A	N/A	0.995
Obstetrical history: high risk	0.817	0.110	4.231	0.819
Obstetrical history: extremely high risk	2.269E-08	N/A	N/A	0.997
Fundal height (3rd trimester)	0.353	0.018	2.233	0.349
Signs of Pre-eclampsia: high risk	8.630	1.713	6.370	0.014^*^
Signs of pre-eclampsia: extremely high risk	1.167E-07	N/A	N/A	0.998
Co-existing conditions: high risk	0.838	0.041	6.132	0.878
Co-existing conditions: extremely high risk	5.199	0.624	31.985	0.086

## Discussion

The CMDS has an AUC of 0.73 for the need of an SBA and 0.85 for mortality, which suggests that it can be used for the purpose of identifying pregnant women who require an SBA prior to delivery and those who are at risk of mortality. The CMDS identified women who required further obstetrical evaluation and skilled care by an SBA.

The CMDS is based on 7 risk factors from the literature that require limited medical knowledge to measure (Table 1). The assessment of pregnant women using the CMDS requires few supplies, all of which are readily available at low cost: a thermometer, measuring tape, blood pressure cuff, height chart, protein and glucose urinalysis strips, and a weight scale for adults.

In this study, signs of pre-eclampsia, obstetrical history, and co-existing conditions were highly associated with the need for an SBA (Table 3). Although the domains of age, parity, fundal height, and BMI were not found to be significantly associated with the need for a SBA at delivery, these factors nonetheless represent important indicators of clinical evaluation that should be considered during routine obstetrical assessment.

Signs of pre-eclampsia was the only risk factor significantly associated with mortality (Table 4). The remaining factors were not significant, which may be attributed to the low absolute number of pregnant women who died in-hospital (n = 7). The estimate of the maternal mortality ratio of 1189 deaths per 100,000 live births is greater than the Nigerian national ratio of 917 deaths per 100,000 live births [1], and similar to previous estimates for Benue State from 2018. [5] Our confidence intervals overlapped with the national estimate, which prevented us from determining if the MMR in Benue State was significantly higher. Future studies with a larger sample size would allow for narrower confidence intervals for MMR in Benue State, as well as sub-group analyses to further examine risk factors for mortality in Benue State.

The CMDS required an interaction term to account for a positive relationship between age and parity with the need for an SBA. Older primigravida and younger multigravida women were more likely to require an SBA than was predicted by these factors alone. Therefore, the prediction of the need for an SBA would be improved by accounting for the interaction between age and parity. In addition, it is possible that the importance of BMI changes in the CMDS criteria was underestimated, as we did not measure perinatal BMI and only reported excessive 3rd trimester weight gain in the pre-eclampsia domain. BMI changes have been noted to be associated with a plethora of maternal and neonatal complications [36], thus future studies on the CMDS should utilize BMI change rather than limiting evaluation to prenatal BMI. This would provide a more accurate AUC estimate in future iterations of the CMDS.

This is the first study in the literature to develop a comprehensive risk analysis tool for all pregnant women at high-risk for mortality and morbidity in low-resource settings. Maternal mortality and morbidity in low-and-middle income countries is disproportionately high, but with targeted interventions such as the one presented, high-risk pregnant women may be more easily identified and encouraged to seek out an SBA in preparation for delivery. The presence of a skilled birth attendant at delivery is critical to ensure a safe, successful delivery for both mother and child. By applying the CMDS in environments where SBA-seeking behaviours are low, SBA uptake may increase which would effectively work towards reducing the high rates of maternal mortality and morbidity in Nigeria and Benue State.

There is the potential for adaptations of the CMDS to identify women at risk and promote improved care, such as by developing the CMDS into a point of care of community screening mobile application with SMS text messages directed to pregnant women who are at high risk for complications to seek an SBA. Indeed, the use of mobile health technologies is rapidly increasing in the African continent [37, 38], and the transformation of a validated scoring system into a mobile application for use by healthcare workers has previously proven successful [39]. Due to the widespread use of smartphones in most African countries, mobile health technologies allow the end-user to virtually access evidence-based resources, such as the CMDS presented here, anywhere and at any time. In the future, we hope to make the CMDS available as a mobile application for use by healthcare personnel in Benue State, Nigeria, as well as pilot the use of directed SMS messaging to promote SBA uptake.

The CMDS was found to hold moderate-to-good discriminative capability within a clinical setting. The major strength of the CMDS is how it is designed for use in low-resource settings by non-specialist healthcare workers. In areas where majority of obstetrical evaluations are done by non-specialists, the CMDS can be used to supplement their assessments of pregnant women during antenatal visits, thus improving care and promoting SBA uptake, ultimately working to reduce maternal morbidity and mortality. We recommend that the CMDS be validated prospectively within a community setting after improvements based on this study have been included. We propose that the CMDS eventually be incorporated into the standard clinical assessment of pregnant women done by non-specialist health workers in low-resource settings.

This study has some limitations. The primary limitation of this study is the limited sample size used in the assessment of the CMDS. This was due to logistical and feasibility constraints, which made it unrealistic to conduct data collection for a longer period of time to obtain a larger sample. An additional limitation is that the CMDS was assessed using a hospital cohort of patients, who were more likely to require an SBA. The CMDS algorithm was unable to account for over 20% of the variability associated with the need for an SBA, thereby suggesting that there are other latent risk factors not captured by the literature search. Thus, future research is required to both identify and include these relevant factors in the CMDS, and prospectively validate the algorithm on a cohort of pregnant women within the community to determine if care is improved and skilled birth attendant utilization is increased.

## Conclusion

The CMDS is a low-cost, evidence-based tool using 7 risk factors assessed in Makurdi. Non-specialist healthcare workers can use the CMDS to encourage pregnant women to seek an SBA in preparation for delivery, thus improving care in low-and-middle-income countries with high rates of maternal mortality and morbidity. Many of the factors assessed by the CMDS closely align with best-practice guidelines for healthcare workers providing obstetrical care to women in rural areas. Altogether, the purpose of this tool is to improve care by health professionals and equip women with the information they need to support their pregnancies, while also mitigating risk through novel applications of widely available mobile technology. The CMDS has potential within the field of maternal care, and further field-testing of the tool will support its widespread implementation. We recommend that further examination of the CMDS in different care settings, including rural, urban, and suburban contexts, be conducted to support its implementation.

## Data Availability

The datasets used and/or analysed during the current study are available from the corresponding author on reasonable request.

## Abbreviations

AUC: Area under the curve
BMI: Body-mass index
CMDS: Community maternal danger score
HIV: Human Immunodeficiency virus
ROC: Receiver operating curve
SBA: Skilled birth attendant
WHO: World Health Organization
95%CI: 95% Confidence interval
